# Multiple Beneficial Effects of AR1001 on Aβ‐Induced Changes in Human Minibrain

**DOI:** 10.1002/alz70861_108692

**Published:** 2025-12-23

**Authors:** Fred Kim, Hyunji Jo, Hansang Cho, Ju‐Suk Nam, Dong‐Keun Song, Kee‐Chan Ahn, Jai Jun Choung

**Affiliations:** ^1^ AriBio Co., Ltd., Seongnam‐si, Gyeonggi‐do Korea, Republic of (South); ^2^ Institute of Quantum Biophysics, Sungkyunkwan University, Suwon, Gyeonggi‐do Korea, Republic of (South)

## Abstract

**Background:**

Human minibrain is an effective model for studying drug effects on neurodegenerative diseases like AD. AR1001, a phosphodiesterase‐5 (PDE5) inhibitor, is being tested in a global Phase 3 clinical trial for early AD at a dosage of 30 mg QD (NCT05531526). This study aims to evaluate effects of AR1001 in the minibrain model.

**Method:**

Three models of human minibrain were used: single‐cultured astrocytes, single‐cultured microglia, and a triculture (neurons, astrocytes and microglia). In the single‐culture models, cells were seeded on day 1 and treated with oAβ (1μM) and IFN‐γ, with or without AR1001 (0.1, 1, and 10μM), from day 0 to day 2. Analyses using immunohistochemistry, ELISA, and immunofluorescence microscopy were conducted on day 2. In the tri‐culture model, neural precursor cells in the central compartment were differentiated for 3 weeks, followed by AR1001 treatment every 2 days for 6 weeks. Microglia were added to the annular compartment, and experiments concluded 4 days later.

**Result:**

In single‐cultured astrocytes, AR1001 significantly enhanced oAβ clearance at 1 and 10μM reducing oAβ–induced inflammatory markers, such as C3 (10μM), NO (0.1, 1, and 10μM), and H_2_O_2_ (1 and 10μM). In single‐cultured microglia, AR1001 notably increased microglia recruitment (10μM) and oAβ clearance (0.1, 1, and 10μM), while decreasing inflammatory markers like CD86 (0.1, 1, and 10μM) and NO (10μM), and elevating the anti‐inflammatory marker CD206 (0.1, 1, and 10μM). In the tri‐culture model, AR1001 inhibited microglial recruitment into the central compartment (0.1, 1, and 10μM) and reduced Aβ1‐42 in the medium (1 and 10μM). It also reduced CD86 (1 and 10μM) and C3 (0.1, 1, and 10μM), while showing trends of increasing CCL2, CCL5, CXCL1, CXCL10, serpin E1 and C‐GSF, and decreasing IL‐8. Additionally, AR1001 markedly restored synapsin 1 and NeuN levels (1 and 10μM) and inhibited increases in *p* ‐tau181 and *p* ‐tau217 (0.1, 1, and 10μM) induced by oAβ.

**Conclusion:**

We found that AR1001 effectively clears oAβ and exhibits anti‐inflammatory effects in astrocytes and microglia. It also provides significant protective effects in neurons, reducing *p* ‐tau levels. These findings highlight the multiple beneficial effects of AR1001 on key brain components: astrocytes, microglia, and neurons.

